# Projective technique “Bird’s Nest Drawing” in child clinical psychology

**DOI:** 10.1192/j.eurpsy.2024.960

**Published:** 2024-08-27

**Authors:** M. Zvereva, N. Zvereva, A. Sergienko, S. Strogova, D. Klak, E. Antonova, E. Balakireva

**Affiliations:** ^1^clinical psychology; ^2^child psychiatry, FSBSI MHRC, Moscow, Russian Federation

## Abstract

**Introduction:**

The “Bird’s Nest Drawing” technique is one of expressive drawing projective techniques. In Russia it has been used since the 2020s. We suggest the pilot version of using this projective technique in child clinical psychology. There were investigated the cognitive and emotional components of performance by children with different types of ontogenesis.

**Purpose:** pilot application of the technique of “Bird’s Nest Drawing” (BND) in the psychological diagnosis of children with different types of ontogenesis.

**Objectives:**

69 children and adolescents 6-17 years old (28 male), examined at the Mental Health Research Center. 1. hospital patients (11-16 years old, 18 persons) diagnoses F20.8, F21.4, 2) outpatient clients with psychological diagnostics (7-16 years old, 45 persons), most of them have psychiatric diagnoses and some of them came for a consultation independently of doctors (there were family and behavior problems). 3) children conceived with the help of assisted reproductive technologies (IVF) - participants in a program for studying cognitive and emotional-personal development (5-13 years old, 8 people). Control group of normal children (14 persons).

**Methods:**

Bird’s Nest Drawing (D.Kaiser, 2003, Kuftyak, 2021) - clinical expert assessment of the drawing parameters (size, location, quality, compliance with instructions) and the emotional component (color, self-assessment of the drawing).

**Results:**

**Table 1.** Frequency of different indicators of BND in compared groups of children.

Groups	N	Central place	High Quality of drawing	Nest out of tree	birds /eggs	Different colors	Safety of nest	High Assessment by participants	High positive emotional expression Assessment by experts
Hospital patients	18 (4 m)	12	4	10	4	10**	4	13**	5**
Outpatient clients	45 (22 m)	38	13	19	17	32	10	25**	17
Children IVF	8 (2 m_	7	2	4	3	6**	1	6	3
Normal children	14(6 m)	14	3	7	4	12**	4	13**	11**

** - significance of the differences (p≤0,05 by φ criterion).

Based on the data obtained, it can be noted that the most different between groups turned out to be an indicator for using different colors, subject’s assessment his picture and emotional expression assessment of picture by experts. Other parameters are similar: please of drawing,

**Discussion:**

We have obtained our own resalts about BND method in children with different type of ontogenesis. These data are similar to D.Kaiser and E.Kuftyak in opportunity of good diagnostic practice of BND method in children and adolescents with different type of ontogenesis in scientific

**Conclusions:**

BND method is a good test for child clinical psychology as a projective one. Restrictions of this investigation - small groups, simple parameters for assessment. We plan to continue this work with more clinical (diagnosis, syndrome et cetera) and sex and age characteristics.

**Disclosure of Interest:**

None Declared

